# Supplementary material for: Healthcare providers’ pain management practice and its associated factors in Ethiopia: A systematic review and meta- analysis
Source: PLoS One. 2024 Nov 20;19(11):e0309094. doi: 10.1371/journal.pone.0309094 (PMC11578494; doi:10.1371/journal.pone.0309094)
Supplement: S2 File — (DOCX) [file pone.0309094.s002.docx]

S2, File. Comprehensive search strategy for healthcare providers’ pain management practice and its associated factors in Ethiopia.Systematic review and meta-analysis

| Databases | Key search terms or phrases |
| --- | --- |
| PubMed | ((("Healthcare providers ") AND ("pain")) AND ("prevalence or incidence")) AND ("factors") AND Ethiopia |
| Total articles | 1000 |
| Cochrane | "(Healthcare providers OR professionals) AND (pain management practice) |
| Total articles | 390 |
| Google | Burden of healthcare providers pain management practice and Ethiopia |
| Total articles | 17 |
| Google Scholar | " prevalence or incidence" pain management practice and Ethiopia |
| Total articles | 500 |
| SCOPUS | Healthcare providers pain management practice in Ethiopia |
| Total Articles | 258 |
| Web of Science | Health care providers OR Nurses OR midwives OR Anesthesia OR General practitioners OR specialists OR Integrated emergency surgical officers AND Ethiopia AND pain management AND incidence AND Ethiopia |
| Total articles | 36 |
| Global Health | Health care providers AND pain management practice And Ethiopia |
| Total Articles | 7 |
| **Total articles retrieved from all databases** | 2208 |
