# Supplementary material for: Healthcare providers’ pain management practice and its associated factors in Ethiopia: A systematic review and meta- analysis
Source: PLoS One. 2024 Nov 20;19(11):e0309094. doi: 10.1371/journal.pone.0309094 (PMC11578494; doi:10.1371/journal.pone.0309094)
Supplement: Attachment — Submitted filename: Comments to the authors on pain managment paractice.docx [file pone.0309094.s003.docx]

Comments for the author

Dear PLoS ONE team of editorials

Thank you for the chance given to me to review a manuscript entitled “Healthcare providers’ pain management practice and its associated factors in Ethiopia. Systematic review and meta- analysis: manuscript number “PONE-d-24-12823”. Since pain management is a fundamental aspect of patient care and quality of life, the finding of the manuscript in general has great clinical significance by providing information on the overall pictures of pain management status in Ethiopia and on the areas of improvement to enhance pain management practice of healthcare providers. The study upholds rigorous technical standards across statistical analyses, and methodological procedures. Please find my general and specific comments below.

**General comments**

- The manuscript has multiple language usage flaws including punctuations, wordings, spelling and mainly grammar errors. These problems are found throughout the manuscript. . Therefore, please make repeated proof-reading and thorough copyediting before considering the manuscript for publication. This would help increase the readability of the manuscript if published..

For instance

- Abstract: Pain defined need to be corrected as pain is defined as
- Under treatment need to be corrected as under-treatment
- Avoid unnecessary spelling in the following sentence. Advancing a national agenda to reduce inconsistencies in pain **C**are for health policy, education and **P**ractice is very imperative
- Inconsistencies in language usage e.g health care professionals’’ versus health care providers’’????
- Work load need to be written as Workload

**Specific comments**

**1. Title**

- The title needs little modification as follows. Healthcare providers’ pain management practice and its associated factors in Ethiopia: A systematic review and met analysis

**2. Key words**

- I recommended the following as a key words. Pain management practice, health care providers, Ethiopia, Systematic review and meta-analysis

**3. Abstract**

- The paragraph stating about the factors associated with the outcome variable need to be rewrite to make it legible and easily comprehended
- The following conclusion is not supported by your finding. “Advancing a national agenda to reduce inconsistencies in pain Care for health policy, education and Practice is very imperative

**4. Introduction**

- The introduction to shall be concentrated on health care providers pain management practice (definition, the figures on magnitude and the inconsistencies in the magnitude, the consequences of poor pain management in terms of quality of life , treatment outcome, healthcare cost…)
- It would be better to add more information significance of the study with regard to the policy makers ( the significance of providing overall figures of pain management practice) , healthcare providers ( training and capacity building activities ) and patients ( in terms of reducing healthcare costs, hospitalization, improving quality of life…)

**5. Methods and materials**

- Word in their first appearance in the manuscript need to be stated in the full explanation terms. GPs, IESO ..
- On the Operational definition, it is better to add about the tool, the assessment criteria ..that your primary studies were utilized.

6. Result and Discussion

- The discussion need to specifically stated for each independent variables
- Please add the further implication of the finding (both on the prevalence and each associated factors ) in the discussion section.
- Please refine the following paragraph accordingly. “The results of this review and meta- analysis identified that availability of pain management protocol and analgesia[**76**], educational level, female sex, took training, work load, work experience, type of profession, attitude and knowledge of health care providers towards pain management practice were significantly associated with pain management practice among health care providers. This results consistent with previous studies conducted in Brazil, USA, Saudi Arabia and Ethiopia [77-81]. As previous research has concluded the practice of having health care providers’ pain management strategies for the patients’ can be categorized into three different models, the biomedical model, the non-pharmacological interventions model, and the alternative medicine model, including acupuncture[82-84],the identified factors are pivotal for advancing pain management practice level of health care providers. Training increase job satisfaction and morale that improve productivity and earn more profit which in turn advances

practicing level of health care providers’ pain management.

Thank you
