# Supplementary material for: Healthcare providers’ pain management practice and its associated factors in Ethiopia: A systematic review and meta- analysis
Source: PLoS One. 2024 Nov 20;19(11):e0309094. doi: 10.1371/journal.pone.0309094 (PMC11578494; doi:10.1371/journal.pone.0309094)
Supplement: Attachment — Submitted filename: Comments to the Author.docx [file pone.0309094.s004.docx]

**Comments to the Authors**

Journal name: PLOS ONE
Manuscript number: PONE-D-24-12823
Title: "HEALTHCARE PROVIDERS’ PAIN MANAGEMENT PRACTICE AND ITS ASSOCIATED FACTORS IN ETHIOPIA. SYSTEMATIC REVIEW AND META-ANALYSIS "

Thank you, editor, for the opportunity to review this manuscript. It addresses the important issue of pain management practice. This issue is critical, given its impact on improving the quality of care and patient satisfaction.

Overall comments: 
The manuscript’s strengths include the study of pain management practice and associated factors using a systematic review and meta-analysis. A specific strength was the relatively well-documented and presented data analysis performed in the "Results section." The weaknesses unfortunately concern the methodology. There are many aspects that compromise the reliability and replicability of the data. Also, the manuscript is full of grammatical errors. The manuscript requires a grammatical and language edition. Thank you.

Major comments:

Abstract

1. The method section of the abstract lacks important details, such as other analysis techniques conducted, such as publication bias and sensitivity analysis. Additionally, it hasn’t included any brief descriptions of the assessment of associated factors.
2. Which guideline was followed for this systematic review and meta-analysis? It should be mentioned.
3. What method was used to pool the prevalence and pool factors? Such as the “Freeman-Tukey double arcsine method." It should be mentioned.
4. In the result section of the abstract, the authors stated a pooled healthcare providers’ pain management practice prevalence of 39.62%. Is it a good or poor practice for pain management? Please make it clear.
5. Factors are not clearly described. For example, what category of education level, knowledge, attitude, workload, and work experience?
6. Why are there only limited searches in some databases? Why didn’t your search include databases such as Embase, CINHAL, and AJOL?
7. The conclusion is difficult to understand. I recommend writing it this way: The prevalence of good practice of pain management among healthcare providers in Ethiopia is low…
8. When saying "poor,” you should try to indicate the comparators. Is there a national target or comparison with other countries?

Introduction

1. The way citations are put in the manuscript is inconsistent. sign.[2]w, regularly[3], on occasion [5]. Some came after a full stop, some before a full stop. Additionally, some have a space before the citation, while others are put without a space. Please put a space before the citation, and citations should be put before a full stop.
2. “An extensive evaluation of previous treatments for pain, which could be the most helpful phase in determining the best course of action for the patient[7].” This statement is not clear.

 - Many of the paragraphs raise redundant ideas. The authors should try to condense similar ideas with in a similar paragraph.

3. “Any kind of pain lowers a person's quality of life[8].” Avoid redundant statements. The previous paragraph indicated that pain diminishes quality of life.
4. The background section failed to include many important issues. Specifically, it didn’t include supportive numerical data, which can show the magnitude of the problem across the globe and in similar contexts. Furthermore, potential factors are not mentioned based on previous study findings. Generally, what is known about the problem is not supported by sufficient evidence.
5. “Various primary reviewed studies on non-pharmacologic and pharmacologic pain management strategies in settings with limited resources.” This statement is unclear and incoherent.
6. “Nevertheless, the researchers did not incorporate meta-analysis-based pooled prevalence estimations.” Not including or incorporating meta-analysis, or meta-analysis not being previously conducted, cannot be the sole reason to conduct a meta-analysis. The authors should come up with a strong gap to conduct a meta-analysis.
7. “Therefore, the current systematic review and meta-analysis address this gap because of the volume of primary evidence that is already available.” What the authors intended to indicate here is not clear at all.
8. “The objective of this study was to look at the combined prevalence of pain management practices used by healthcare providers and the factors that are related to it among Ethiopians.” Please shorten this statement. “Healthcare providers in Ethiopia” and “Ethiopians” have a huge difference. Including the term “Ethiopians” will refer to the inclusion of study participants who are Ethiopian citizens only. What about foreigners working in Ethiopian health care? Please avoid misleading statements and stick with the title.

Method

1. “To determine the pooled prevalence and associated factors of pain management practice among Ethiopian healthcare providers, a systematic review and meta-analysis were carried out.” The authors should understand the difference between “Ethiopian healthcare providers” and “healthcare providers in Ethiopia”. The first indicates any healthcare provider across the globe who is Ethiopian, while the second indicates any healthcare provider in Ethiopia without considering nationality. The use of those phrases interchangeably creates ambiguity as well as deviations from the title.
2. Please include a supplementary file of the PRISMA check list used.
3. The study designs need to be specified in the databases and search strategy sections. The manuscript indicated the authors searched to find all primary studies. Did this study include case reports, case series, and case studies too?
4. The number as well as the types of databases searched significantly vary in the abstract and method sections. Why? Based on the method section, only 4 databases are searched, which might limit the number of potential studies to be included.
5. Some of the search terms and phrases are not MeSH terms. Any reasons for using free text searches?
6. “health care providers” was used as one search term. However, throughout the document, “healthcare” is used, and it is not included as a search term.
7. Any specific reason to take 2015 as the study inception point?
8. As eligibility criteria, the criteria used before including the studies should be mentioned.
9. “The review included all observational (cross-sectional) studies that were written in English.” Why only include cross-sectional studies? The second objective of this study is to identify associated factors. Hence, why did the authors exclude case control and other study designs?
10. I recommend rewriting the eligibility section by clearly describing: i) study period: ii) study designs: iii) population; iv) outcome; v) place of study: studies conducted in Ethiopia; and vi) studies published in the English language. However, citations without abstracts, full texts, anonymous reports, editorials, systematic reviews, meta-analyses, and qualitative studies were excluded from the review.
11. Since this study included observational studies, please include the CoCoPop framework (condition, context, and population).
12. For the study selection, an odd number of authors should review the eligibility. In your case, a third author could have been used as a tiebreaker.
13. “The third reviewer (WN) was involved while the differences between the data extractors persisted.” The word while should be replaced with when.
14. On the data extraction, some pertinent variables are not extracted, such as population. Any reasons for not extracting the population? If there is no strong reason, I recommend including the population, as it will help in the subgroup analysis.

15. 15. The main outcome of interest is not clear for me. If it is a binary outcome, the categories should be mentioned.

16. I don’t think including an operational definition is important, as the included studies might have used different criteria. This should be mentioned in the characteristics of the included studies section.
17. “We used Epi-Info in conjunction with descriptive statistics to translate Risk Ratio data from the included primary studies into odds ratios (ORs).” The included studies are all cross-sectional, and if that is the case, where did risk ratios come from? Odds ratios are reported in cross-sectional studies with a binary outcome variable.
18. What type of method was used to conduct the sensitivity analysis? Please be specific and mention it.
19. Details regarding the subgroup analysis conducted were not mentioned in the data analysis section of the method part.
20. How was the quality of the included studies assessed? The study did not include a quality assessment section in the method section. This will significantly affect the quality of the systematic review and meta-analysis.

Results

1. “eight studies in Southern nations nationalities[32, 35, 50, 53, 59-61]”. The cited articles are seven.
2. Based on geographical region, 36 studies are specified; what about the remaining two studies?
3. “ The total sample size of the included studies was 283,572,” This statement is completely wrong.
4. All table and figure captions should be self-explanatory. For example, figure 1’s caption is not self-explanatory.
5. Table 1: The year should be displayed in a separate column. As all studies are cross-sectional, mentioning the study design in the table is less relevant.
6. There were no methodological mentions regarding quality appraisal. However, Table 2 indicates quality appraisal. This is incoherent and should be appropriately mentioned.
7. The inception time for the included studies was 2015. But one study conducted in 2014 was included. Any justifications?
8. The description mentioned about the quality appraisal should be placed in the method section, not in the result section.
9. Sensitivity Exploration: We conducted a sensitivity analysis to determine the impact of a specific study on the overall meta-analysis. The estimate from a single study is then closer to the pooled estimate, as seen by the forest plot, suggesting that there is no effect of a single study on the overall pooled estimate.” Statements explaining the methodology should not be included in the result section. Just show and clarify the results.
10. Please provide a general heading for the sub-group analysis (sub-group analysis) with a text description too, and then provide subheadings based on the criteria used for sub-group analysis. For example, sub-group analysis using the methods of pain management
11. “This finding revealed that non-pharmacological pain management practice of health care professionals slightly greater than pharmacological pain management practice of health care providers.” By observing the confidence intervals of the two pain management techniques, it can be understood that the pooled prevalence of one technique falls in between the confidence intervals of the second technique. Considering this, you cannot say one technique is slightly greater than the other. The same comments apply to the sub-group analyses based on pain types and sample size too.
12. Generally, the sub-group analyses conducted based on the three criteria failed to find the possible sources of the significantly high heterogeneity. I recommend conducting further sub-group analyses based on years of publication and other factors.
13. The characteristics of the included studies should include descriptions regarding the criteria used in the sub-group analysis. Otherwise, readers will find it difficult to understand the sub-group analysis. For example, there was no prior description of how many studies assessed pharmacological or non-pharmacological methods. Similarly, this comment applies to the types of pain too.

-Discussion

-1. Please be specific when mentioning the outcome variable. “The overall pooled prevalence of pain management practices among healthcare providers in this study was 39.62% (95% CI: 34.78, 44.47); I2=97.00%; P<0.000”. Specify which category you are referring to (good practice vs. poor practice). Consider this comment for the whole manuscript.

2. The discussion section is too shallow, and it needs rewriting. The discussion should include an introduction and follow the format of: what your study aimed to do, what your study found, how these findings compare with other literature, the possible justifications for the similarities or differences with possible supportive scientific evidence and citations, and how these findings can be adapted in real-life practice or implications. Each section forming a paragraph. You do include some of these points, but they are not sufficient as they currently stand.

Strengths and limitations of the study

1. Sample size “(n=283,572)”. It is unreal and misleading. Please add the sample sizes of the included studies and put the correct number.
2. The limitations are not well stated and explained. Please explain the limitations in detail. You can consider the limitations mentioned by the included studies.

Tables and figures

1. The total number of tables and figures in combination should not be more than 7. For associated factors, use a summary table and remove all the figures related to the factors.
2. Figure 1: Use the PRISMA 2020 flow diagram. The one you used is the PRISMA 2009 flow diagram.

Minor comments:

Author names: ShegawZeleke. Please insert a space.
Key words: Please use MeSH terms as key words. For example, “practice” is not a MeSH word. Put “systematic review and meta-analysis” separately.

Abstract

1. Include the objective of the study as a therefore statement in the introduction section of the abstract.

2. “The procedure of this systematic review and meta-analysis was registered at Prospero under registration number CRD42024512855”. This statement is not that significant to be included in the abstract. It would be better to delete it from the abstract, as it is sufficient if only mentioned in the method section of the manuscript.

3. “educational level(AOR=3.3, 95%CI: 2.53, 4.13)” A space should be used before inserting a parenthesis.

4. I recommend using a single digit decimal point.

5. “ The prevalence of health care providers’ pain management practice was obtained from thirty eight included primary studies [13, 20, 29- 56].Which ranges from 13-66.” Indicate the numbers as percent (13-66%). Additionally, cite the references.

6. The publication year is not mentioned for one included study (Ganta. M, et al.,). Please include a publication year.

7. Publication bias among the studies: “Thus, in order to control the publication bias, we did not perform trim and fill analysis.” There is no need to include this statement, as the result of the funnel plot did not need a trim and fill analysis.

**Generally:** based on the current status of the manuscript, it needs an extensive revision. Additionally, the manuscript is full of grammatical and typographical errors. As a result, the manuscript needs language editing from an editorial service or from fluent English-speaking colleagues. Thank you, and good luck!
