# Supplementary material for: Healthcare providers’ pain management practice and its associated factors in Ethiopia: A systematic review and meta- analysis
Source: PLoS One. 2024 Nov 20;19(11):e0309094. doi: 10.1371/journal.pone.0309094 (PMC11578494; doi:10.1371/journal.pone.0309094)
Supplement: Attachment — Submitted filename: Authors response t o Editeres and reviwers and .docx [file pone.0309094.s005.docx]

**Author’s Point-by-Point Response to the Editors and Reviewer's comments**

PONE-D-24-12823

HEALTHCARE PROVIDERS’ PAIN MANAGEMENT PRACTICE AND ITS ASSOCIATED FACTORS IN ETHIOPIA. SYSTEMATIC REVIEW AND META- ANALYSIS

PLOS ONE

Dear Dr. Mekonnen,

Thank you for submitting your manuscript to PLOS ONE. After careful consideration, we feel that it has merit but does not fully meet PLOS ONE’s publication criteria as it currently stands. Therefore, we invite you to submit a revised version of the manuscript that addresses the points raised during the review process.

Please submit your revised manuscript by Aug 01 2024 11:59PM. If you will need more time than this to complete your revisions, please reply to this message or contact the journal office at [plosone@plos.org](mailto:plosone@plos.org). When you're ready to submit your revision, log on to https://www.editorialmanager.com/pone/ and select the 'Submissions Needing Revision' folder to locate your manuscript file.

Please include the following items when submitting your revised manuscript:

- A rebuttal letter that responds to each point raised by the academic editor and reviewer(s). You should upload this letter as a separate file labeled 'Response to Reviewers'.
- A marked-up copy of your manuscript that highlights changes made to the original version. You should upload this as a separate file labeled 'Revised Manuscript with Track Changes'.
- An unmarked version of your revised paper without tracked changes. You should upload this as a separate file labeled 'Manuscript'.

If you would like to make changes to your financial disclosure, please include your updated statement in your cover letter. Guidelines for resubmitting your figure files are available below the reviewer comments at the end of this letter.

If applicable, we recommend that you deposit your laboratory protocols in protocols.io to enhance the reproducibility of your results. Protocols.io assigns your protocol its own identifier (DOI) so that it can be cited independently in the future. For instructions see: <https://journals.plos.org/plosone/s/submission-guidelines#loc-laboratory-protocols>. Additionally, PLOS ONE offers an option for publishing peer-reviewed Lab Protocol articles, which describe protocols hosted on protocols.io. Read more information on sharing protocols at <https://plos.org/protocols?utm_medium=editorial-email&utm_source=authorletters&utm_campaign=protocols>.

We look forward to receiving your revised manuscript.

Kind regards,

Dereje Zewdu Assefa, BSc, MSc
Academic Editor
PLOS ONE

**Point by point response for editors (the responses are written in BOLD)**

**First of all, the authors would like to thank the Journal editors and the respective reviewers for reviewing our manuscript and providing you with all the necessary comments to be considered. As per the feedback, we responded point by point. The authors tried to answer all the issues raised by the editorial team and reviewers.**

Journal Requirements:

1. When submitting your revision, we need you to address these additional requirements.

Please ensure that your manuscript meets PLOS ONE's style requirements, including those for file naming. The PLOS ONE style templates can be found at 
https://journals.plos.org/plosone/s/file?id=wjVg/PLOSOne_formatting_sample_main_body.pdf and 
[**https://journals.plos.org/plosone/s/file?id=ba62/PLOSOne_formatting_sample_title_authors_affiliations.pdf**](https://journals.plos.org/plosone/s/file?id=ba62/PLOSOne_formatting_sample_title_authors_affiliations.pdf)

**Authors’ response: Thank you so much for your valuable comments and suggestions.** **We have carefully reviewed the PLOS ONE style templates provided and ensured that our manuscript complies with all specified formatting guidelines, including those for file naming.**

2. We note that your Data Availability Statement is currently as follows: All relevant data are within the manuscript and its Supporting Information files.

Please confirm at this time whether or not your submission contains all raw data required to replicate the results of your study. Authors must share the “minimal data set” for their submission. PLOS defines the minimal data set to consist of the data required to replicate all study findings reported in the article, as well as related metadata and methods (https://journals.plos.org/plosone/s/data-availability#loc-minimal-data-set-definition).

For example, authors should submit the following data:

- The values behind the means, standard deviations and other measures reported;
- The values used to build graphs;
- The points extracted from images for analysis.

Authors do not need to submit their entire data set if only a portion of the data was used in the reported study.

If your submission does not contain these data, please either upload them as Supporting Information files or deposit them to a stable, public repository and provide us with the relevant URLs, DOIs, or accession numbers. For a list of recommended repositories, please see https://journals.plos.org/plosone/s/recommended-repositories.

If there are ethical or legal restrictions on sharing a de-identified data set, please explain them in detail (e.g., data contain potentially sensitive information, data are owned by a third-party organization, etc.) and who has imposed them (e.g., an ethics committee). Please also provide contact information for a data access committee, ethics committee, or other institutional body to which data requests may be sent. If data are owned by a third party, please indicate how others may request data access.

**Authors’ response:** **Thank you for your message and for highlighting the requirements regarding the Data Availability Statement for our submission.**

**We confirm that our manuscript currently includes all relevant data necessary to replicate the study findings, as stated in our Data Availability Statement. However, upon your request, we are prepared to provide additional details and ensure compliance with PLOS ONE's guidelines for the minimal data set.**

**Please find attached the necessary data files, including:**

- **The values behind the means, standard deviations, and other reported measures.**
- **The data used to generate graphs.**
- **Extracted points from images used for analysis.**

**These files are intended to facilitate replication of our results and can be considered as the minimal data set required**

3. Please amend either the abstract on the online submission form (via Edit Submission) or the abstract in the manuscript so that they are identical.

**Authors’ response:** **Thank you for your concern for bringing this to our attention. We apologize for the oversight regarding the abstract discrepancy. We have now reviewed both the abstract on the online submission form and the abstract within the manuscript, and have ensured they are identical.**

4. Please include captions for your Supporting Information files at the end of your manuscript, and update any in-text citations to match accordingly. Please see our Supporting Information guidelines for more information: http://journals.plos.org/plosone/s/supporting-information.

**Authors’ response:** **Thank you for your guidance regarding the inclusion of captions for supporting information files. We have reviewed the Supporting Information guidelines as per your suggestion and ensured that captions for all Supporting information files are included at the end of our manuscript. Additionally, we updated any in-text citations accordingly to match the order and content of the Supporting information (Please find the revised manuscript attached).**

Additional Editor Comments:

"The manuscript needs thorough language editing and proofreading." Please remember to add the search strategy as a supplementary file and provide details of the electronic database used, along with the corresponding article numbers found in each database.


[Note: HTML markup is below. Please do not edit.]

**Authors’ response:** **Thank you for your feedback and suggestions for improving our manuscript. We tried thorough language editing and proofreading, and we ensured that these aspects addressed comprehensively (Please see the revised manuscript). Regarding the search strategy, we included the search strategy as a supplementary file in the revised submission (See supplementary file –2).**

Reviewers' comments:

Reviewer's Responses to Questions

**Comments to the Author**

1. Is the manuscript technically sound, and do the data support the conclusions?

The manuscript must describe a technically sound piece of scientific research with data that supports the conclusions. Experiments must have been conducted rigorously, with appropriate controls, replication, and sample sizes. The conclusions must be drawn appropriately based on the data presented.

Reviewer #1: Yes

Reviewer #2: Partly

2. Has the statistical analysis been performed appropriately and rigorously?

Reviewer #1: Yes

Reviewer #2: Yes

3. Have the authors made all data underlying the findings in their manuscript fully available?

The [PLOS Data policy](http://www.plosone.org/static/policies.action#sharing) requires authors to make all data underlying the findings described in their manuscript fully available without restriction, with rare exception (please refer to the Data Availability Statement in the manuscript PDF file). The data should be provided as part of the manuscript or its supporting information, or deposited to a public repository. For example, in addition to summary statistics, the data points behind means, medians and variance measures should be available. If there are restrictions on publicly sharing data—e.g. participant privacy or use of data from a third party—those must be specified.

Reviewer #1: Yes

Reviewer #2: Yes

4. Is the manuscript presented in an intelligible fashion and written in standard English?

PLOS ONE does not copyedit accepted manuscripts, so the language in submitted articles must be clear, correct, and unambiguous. Any typographical or grammatical errors should be corrected at revision, so please note any specific errors here.

Reviewer #1: No

Reviewer #2: No

5. Review Comments to the Author

Please use the space provided to explain your answers to the questions above. You may also include additional comments for the author, including concerns about dual publication, research ethics, or publication ethics. (Please upload your review as an attachment if it exceeds 20,000 characters)

**Point by point response for Reviewers (the responses are written in BOLD)**

**First of all, the authors would like to thank the Journal editors and the respective reviewers for reviewing our manuscript and providing you with all the necessary comments to be considered. As per the feedback, we responded point by point. The authors tried to answer all the issues raised by the editorial team and reviewers.**

**Reviewer #1:** Comments for the author
Dear PLoS ONE team of editorials
Thank you for the chance given to me to review a manuscript entitled “Healthcare providers’ pain management practice and its associated factors in Ethiopia. Systematic review and meta- analysis: manuscript number “PONE-d-24-12823”. Since pain management is a fundamental aspect of patient care and quality of life, the finding of the manuscript in general has great clinical significance by providing information on the overall pictures of pain management status in Ethiopia and on the areas of improvement to enhance pain management practice of healthcare providers. The study upholds rigorous technical standards across statistical analyses, and methodological procedures. Please find my general and specific comments below.

**Authors’ response: Thank you for taking the time to provide such detailed feedback on our manuscript. We genuinely appreciated your thorough review and the constructive suggestions you've offered to improve the impact and quality of our work. Your insights are invaluable to us, and we carefully considered each comment and recommendation in the revision process**

General comments
ϖ The manuscript has multiple language usage flaws including punctuations, wordings, spelling and mainly grammar errors. These problems are found throughout the manuscript. . Therefore, please make repeated proof-reading and thorough copyediting before considering the manuscript for publication. This would help increase the readability of the manuscript if published..

For instance
⎫ Abstract: Pain defined need to be corrected as pain is defined as
⎫ Under treatment need to be corrected as under-treatment
⎫ Avoid unnecessary spelling in the following sentence. Advancing a national agenda to reduce inconsistencies in pain Care for health policy, education and Practice is very imperative
⎫ Inconsistencies in language usage e.g health care professionals’’ versus health care providers’’????
⎫ Work load need to be written as Workload

**Authors’ response:** **Thank you for your in-depth review and valuable feedback on our manuscript.as much as we can we tried to improve punctuation, wording, spelling, and grammar errors throughout the manuscript.** **Specifically based on your comment: Correct "Pain defined" to "Pain is defined as" in the Abstract, Change "Under treatment" to "Under-treatment**."**, Ensured consistency of "health care professionals" versus "health care providers."**, **Correct "Work load" to "Workload." and revised the sentence to avoid unnecessary spelling and improve clarity: "Advancing a national agenda to reduce inconsistencies in pain care for health policy, education, and practice is imperative."**
Specific comments
1. Title
ϖ The title needs little modification as follows. Healthcare providers’ pain management practice and its associated factors in Ethiopia: A systematic review and met analysis

**Authors’ response:** **Thank you for your feedback regarding the title of our manuscript.** **Based on your recommendation, we revised title: "Healthcare Providers’ Pain Management Practice and Its Associated Factors in Ethiopia: A Systematic Review and Meta-Analysis"**
2. Key words
⎫ I recommended the following as a key words. Pain management practice, health care providers, Ethiopia, Systematic review and meta-analysis

**Authors’ response:** **Thank you for your recommendations regarding the keywords for our manuscript.** **Based on your suggestion, we have included Pain management practice, Healthcare providers, Ethiopia, Systematic review and Meta-analysis as key words**
3. Abstract
⎫ The paragraph stating about the factors associated with the outcome variable need to be rewrite to make it legible and easily comprehended
⎫ The following conclusion is not supported by your finding. “Advancing a national agenda to reduce inconsistencies in pain Care for health policy, education and Practice is very imperative

**Authors’ response:** **Thank you for your comment on our manuscript.** **Regarding the paragraph discussing factors associated with the outcome variable and the conclusion related to advancing a national agenda. We revised to improve clarity and readability and conclude accurately drawn from our study results. Thank you once again for your valuable suggestion.**
4. Introduction
⎫ The introduction to shall be concentrated on health care providers pain management practice (definition, the figures on magnitude and the inconsistencies in the magnitude, the consequences of poor pain management in terms of quality of life , treatment outcome, healthcare cost…)
⎫ It would be better to add more information significance of the study with regard to the policy makers ( the significance of providing overall figures of pain management practice) , healthcare providers ( training and capacity building activities ) and patients ( in terms of reducing healthcare costs, hospitalization, improving quality of life…)

**Authors’ response:** **Thank you for your insightful feedback to enhance the clarity and relevance of the introduction. Specifically, on**

**1. Defining healthcare providers' pain management practice and highlighting the varying magnitudes and inconsistencies in current practices.**

**2. Exploring the consequences of inadequate pain management in terms of quality of life, treatment outcomes, and healthcare costs and significance of the study by emphasizing for:**

A. **Policy makers, by providing comprehensive figures on pain management practices to guide healthcare policies.**
B. **Healthcare providers, by discussing training and capacity-building activities to enhance pain management skills.**
C. **Patients, focusing on the potential benefits such as reduced healthcare costs, decreased hospitalizations, and improved quality of life.**

**We ensured that these enhancements are incorporated into the revised introduction to provide a clearer and more comprehensive overview of the study’s significance**
5. Methods and materials
⎫ Word in their first appearance in the manuscript need to be stated in the full explanation terms. GPs, IESO ..
⎫ On the Operational definition, it is better to add about the tool, the assessment criteria..that your primary studies were utilized.

**Authors’ response:** **Thank you for your comment and recommendations about how we can use in expand form and abbreviated form particularly GPs and IESO, we fully spelled out upon their first mention in the manuscript. Regarding the operational definition,** **in the revised manuscript, we included additional information on the specific tools and assessment criteria utilized**
6. Result and Discussion
⎫ The discussion need to specifically stated for each independent variables
⎫ Please add the further implication of the finding (both on the prevalence and each associated factors ) in the discussion section.
⎫ Please refine the following paragraph accordingly. “The results of this review and meta- analysis identified that availability of pain management protocol and analgesia[76], educational level, female sex, took training, work load, work experience, type of profession, attitude and knowledge of health care providers towards pain management practice were significantly associated with pain management practice among health care providers. This results consistent with previous studies conducted in Brazil, USA, Saudi Arabia and Ethiopia [77-81]. As previous research has concluded the practice of having health care providers’ pain management strategies for the patients’ can be categorized into three different models, the biomedical model, the non-pharmacological interventions model, and the alternative medicine model, including acupuncture[82-84],the identified factors are pivotal for advancing pain management practice level of health care providers. Training increase job satisfaction and morale that improve productivity and earn more profit which in turn advances
practicing level of health care providers’ pain management.

1. **Authors’ response:** **Thank you for your detailed feedback on the discussion section of our manuscript.** **Here is how we plan to address each of your points: Specificity of Independent Variables: We will ensure that the discussion section clearly specifies the impact and relevance of each independent variable on pain management practice among healthcare providers.**
2. **Further Implications of Findings: We will expand the discussion to include additional implications of our findings, particularly focusing on the prevalence of pain management practices and the implications of each associated factor identified in our study.**
3. **Refinement of Paragraph: We will refine the paragraph as follows to improve clarity and coherence:**

**"The results of this review and meta-analysis indicate significant associations between pain management practice among healthcare providers and several factors, including the availability of pain management protocols and analgesia [76], educational level, sex, training history, workload, work experience, profession type, and attitudes and knowledge towards pain management. These findings are consistent with prior studies conducted in Brazil, USA, Saudi Arabia, and Ethiopia [77-81]. Previous research has categorized healthcare providers' pain management strategies into biomedical, non-pharmacological interventions, and alternative medicine models, including acupuncture [82-84]. The identified factors play a pivotal role in advancing the level of pain management practices among healthcare providers. Training improves job satisfaction and morale, enhancing productivity and ultimately contributing to the advancement of healthcare providers' pain management practices."We ensured that these revisions are integrated into the discussion section to strengthen the manuscript's clarity and coherence.**

Thank you

**Reviewer #2:** Overall comments:
The manuscript’s strengths include the study of pain management practice and associated factors using a systematic review and meta-analysis. A specific strength was the relatively well-documented and presented data analysis performed in the "Results section." The weaknesses unfortunately concern the methodology. There are many aspects that compromise the reliability and replicability of the data. Also, the manuscript is full of grammatical errors. The manuscript requires a grammatical and language edition. Please refer to the attached comments. Thank you.

**Authors’ response: Thank you so much we did as your suggestion point by point from attached comments.**

6. PLOS authors have the option to publish the peer review history of their article ([what does this mean?](https://journals.plos.org/plosone/s/editorial-and-peer-review-process#loc-peer-review-history)). If published, this will include your full peer review and any attached files.


If you choose “no”, your identity will remain anonymous but your review may still be made public.


**Do you want your identity to be public for this peer review?** For information about this choice, including consent withdrawal, please see our [Privacy Policy](https://www.plos.org/privacy-policy).

Reviewer #1: No

Reviewer #2: No
