# Supplementary material for: Healthcare providers’ pain management practice and its associated factors in Ethiopia: A systematic review and meta- analysis
Source: PLoS One. 2024 Nov 20;19(11):e0309094. doi: 10.1371/journal.pone.0309094 (PMC11578494; doi:10.1371/journal.pone.0309094)
Supplement: Attachment — Submitted filename: Comments to the Author.docx [file pone.0309094.s006.docx]

**Comments to the Authors**

Journal name: PLOS ONE
Manuscript number: PONE-D-24-12823R1
Title: “Healthcare providers’ pain management practice and its associated factors in Ethiopia: A systematic review and meta- analysis.”

Thank you, editor, for the opportunity to review this manuscript. It addresses the important issue of pain management practice. This issue is critical, given its impact on improving the quality of care and patient satisfaction.

Overall comments: 
The manuscript’s strengths include the study of pain management practice and associated factors using a systematic review and meta-analysis. A specific strength was the relatively well-documented and presented data analysis performed in the "Results section." Even though there is a significant improvement in the revised manuscript, it still needs a significant revision. Additionally, despite the improvements, grammatical and typographical errors still persist. As a result, the manuscript needs language editing from an editorial service or from fluent English-speaking colleagues. Thank you!

Major comments:

Abstract

In the method part of the abstract, “This systematic review and meta-analysis was followed Preferred Reporting Items for Systematic Reviews and Meta-Analyses guidelines.” Please remove ‘was’.

The authors mentioned the use of logistic regression analysis to pool the odds ratios from different studies. However, I asked specifically how they pooled the logistic regression analysis from different studies. It would be highly important if the authors could specify the specific technique used. One example of a pooling method includes ‘The Mantel-Haenszel method’. You can read further.

The authors have indicated that the overall pain management practice is low compared to other benchmarks. If that is the case, they should mention it in the conclusion part of the abstract.

Write it this way: The prevalence of pain management practice among healthcare providers in Ethiopia is low compared to the national…,that sets a target of …Introduction

3. Put a space before indicating the 30th reference in the introduction section.
4. Even though there is much improvement in the introduction section, the flow of ideas is inconsistent and difficult to grasp. I recommend first mentioning general issues regarding pain management, followed by figure based (data-based)  findings across the globe, then mentioning factors, and closing the section by showing the research gap and what this study will do. (The contents are good, but the paragraphs need reshuffling.)
5. The final paragraph of the introduction section included statements that were outside the scope of this study. This study cannot guarantee the provision of training or reduce healthcare costs.
6. Remove this statement, “which promotes training and capacity building that enhance pain management skills for them in Ethiopia.”
7. Remove this statement, “which initiates for designing appropriate strategies and intervention. Additionally, it reduces healthcare costs, hospitalization and improving quality of life for patients’ in Ethiopia.”

Method

1. Please indicate in the text where the PRISMA checklist can be located.  (Supplementary file 1).
2. "The review included all observational (cross-sectional) studies that were written in English.” Why only include cross-sectional studies? The second objective of this study is to identify associated factors. Hence, why did the authors exclude case control and other study designs? This previous question has not been answered. If there are case control studies on the topic that reported odds ratios, they should be included, as identifying factors is the second objective of this systematic review and meta-analysis.
3. Since this study included observational studies, please include the CoCoPop framework (condition, context, and population). It hasn’t been included in the revised manuscript. Please mention it in the manuscript in a clearer way.
4. “The third reviewer (WN) was involved while the differences between the data extractors persisted.” The word while should be replaced with when.
5. On the data extraction, some pertinent variables are not extracted, such as population. Any reasons for not extracting the population? If there is no strong reason, I recommend including the population, as it will help in the subgroup analysis. The response provided for this question is not satisfactory. I personally believe that there might be differences in practice between different health professionals, for example, medical doctors vs. nurses. That is why I recommended extracting the population so that it can be considered for sub-group analysis.
6. What type of method was used to conduct the sensitivity analysis? Please be specific and mention it. It hasn’t been mentioned. Please mention “one-at-a-time method” in the manuscript.
7. The variables used for the sub-group analysis should be specifically mentioned.
8. On the quality assessment, include all studies and cite them. Currently, only 30 out of the 38 studies are cited [13, 22-26, 38-61].
9. Don’t include results regarding the quality assessment in the method section.

Results

1. Characteristics of the Included Studies: “The prevalence of health care providers’ pain
2. management practice was obtained from thirty-eight included primary studies [13, 22-26, 38-61].” The authors mentioned 38 studies but cited only 30. Why? Please cite all studies.
3. Most of the tables and figure captions are not self-explanatory. For example, Table 1’s caption is not self-explanatory. Please consider this example: Table 1: Characteristics of the included studies for the systematic review and meta-analysis of…The inception time for the included studies was 2015. But one study conducted in 2014 was included. Any justifications? The answer provided is not acceptable at all. Either include 2014 as an inception time or remove the study conducted in 2014.
4. The description mentioned about the items of quality appraisal should be placed in the method section, not in the result section.
5. Sensitivity Exploration: We conducted a sensitivity analysis to determine the impact of a specific study on the overall meta-analysis. The estimate from a single study is then closer to the pooled estimate, as seen by the forest plot, suggesting that there is no effect of a single study on the overall pooled estimate.” Statements explaining the methodology should not be included in the result section. Just show and clarify the results.
6. In the sub-group analysis, there is no need to describe the importance and reasons for conducting a sub-group analysis.
7. Words like “all most” (almost) are grammatically incorrect, and please use terms like 'similar’.
8. For associated factors, most of the statements have grammatical errors, and additionally, the use of brackets varies from factor to factor. A P value of <0.000 is incorrect in the mathematical sense. Please replace it with <0.001.

-Discussion

1. The discussion section is still too shallow and it needs rewriting. You should discuss the variations in prevalence between this study and other studies in a detailed way and add other literature too.

2. Factors should be discussed one by one.

Limitation of the study

The limitations are not well stated and explained. Please explain the limitations in detail. You can consider the limitations mentioned by the included studies.

Conclusion:

Please mention the comparators and the standard when saying low.

Generally, even though there is a significant improvement in the revised manuscript, it still needs a significant revision. Additionally, despite the improvements, grammatical and typographical errors still persist. As a result, the manuscript needs language editing from an editorial service or from fluent English-speaking colleagues. Thank you!
