# Supplementary material for: Healthcare providers’ pain management practice and its associated factors in Ethiopia: A systematic review and meta- analysis
Source: PLoS One. 2024 Nov 20;19(11):e0309094. doi: 10.1371/journal.pone.0309094 (PMC11578494; doi:10.1371/journal.pone.0309094)
Supplement: Attachment — Submitted filename: Response for Reviwer.docx [file pone.0309094.s007.docx]

**Comments to the Authors**

Journal name: PLOS ONE
Manuscript number: PONE-D-24-12823R1
Title: “Healthcare providers’ pain management practice and its associated factors in Ethiopia: A systematic review and meta- analysis.”

Thank you, editor, for the opportunity to review this manuscript. It addresses the important issue of pain management practice. This issue is critical, given its impact on improving the quality of care and patient satisfaction.

**Point by point response for Reviewer (the responses are written in BOLD)**

Overall comments: 
The manuscript’s strengths include the study of pain management practice and associated factors using a systematic review and meta-analysis. A specific strength was the relatively well-documented and presented data analysis performed in the "Results section." Even though there is a significant improvement in the revised manuscript, it still needs a significant revision. Additionally, despite the improvements, grammatical and typographical errors still persist. As a result, the manuscript needs language editing from an editorial service or from fluent English-speaking colleagues. Thank you!

**Authors’ response:** **Thank you very much for your thoughtful and constructive feedback on our manuscript. We appreciate your observation that the manuscript has shown significant improvement and acknowledge your point regarding the need for further revisions. We are committed to addressing the remaining issues to enhance the quality of our manuscript thoroughly review and correct revisions carefully to address any remaining concerns and ensure that the text is clear, precise, and meets the high standards required for publication Thank you once Again!!!(See throughout all revised manuscript).**

Major comments:

Abstract

In the method part of the abstract, “This systematic review and meta-analysis was followed Preferred Reporting Items for Systematic Reviews and Meta-Analyses guidelines.” Please remove ‘was’.

**Authors’ response:** **Thank you for your valuable feedback on our manuscript. We changed it from** **“This systematic review and meta-analysis was followed Preferred Reporting Items for Systematic Reviews and Meta-Analyses guidelines. “To “This systematic review and meta-analysis followed Preferred Reporting Items for Systematic Reviews and Meta-Analyses guidelines (See at abstract section page number-2, line-7).**

The authors mentioned the use of logistic regression analysis to pool the odds ratios from different studies. However, I asked specifically how they pooled the logistic regression analysis from different studies. It would be highly important if the authors could specify the specific technique used. One example of a pooling method includes ‘The Mantel-Haenszel method’. You can read further.

**Authors’ response:** **Thank you for your insightful comment. There are different Techniques Used for Pooling. e.g. Fixed-Effects Model,** **Random-Effects Model,** **Mantel-Haenszel Method,** **DerSimonian and Laird Method which has its own specific significant.** **We used the DerSimonian and Laird random-effects model for combining the odds ratios from different studies. This model is appropriate as it accounts for between-study variability as well as within-study variability. We revised the Methods section to reflect this as “We utilized the DerSimonian and Laird random-effects model to pool the odds ratios across studies, accommodating both within-study and between-study variability.”(See at abstraction section page number 2, line 11).**

The authors have indicated that the overall pain management practice is low compared to other benchmarks. If that is the case, they should mention it in the conclusion part of the abstract.

**Authors’ response:** **Thank you for helping us strengthens the abstract. We rewrote it at abstract conclusion section section last paragraph of Revised Conclusion:**

Write it this way: The prevalence of pain management practice among healthcare providers in Ethiopia is low compared to the national… that sets a target of …Introduction

**Authors’ response:** **Thank you.** **We modified as your recommendation at abstraction section at conclusion part see page number two.**

3. Put a space before indicating the 30th reference in the introduction section.

**Authors’ response:** **Thank you. We have now added a space before the 30th reference in the Introduction section as suggested. ( page 5, line –number -4)**

4. Even though there is much improvement in the introduction section, the flow of ideas is inconsistent and difficult to grasp. I recommend first mentioning general issues regarding pain management, followed by figure based (data-based) findings across the globe, then mentioning factors, and closing the section by showing the research gap and what this study will do. (The contents are good, but the paragraphs need reshuffling.)

**Authors’ response:** **Thank you for your insightful feedback on the Introduction section of our manuscript for the need of reshuffling.** **We have revised the Introduction section to better align with your recommendations.( see introduction section, page number 3 to 5).**

5. The final paragraph of the introduction section included statements that were outside the scope of this study. This study cannot guarantee the provision of training or reduce healthcare costs.

**Authors’ response:** **We have revised the final paragraph of the Introduction to align it more closely with the actual scope and objectives of the study.**

6. Remove this statement, “which promotes training and capacity building that enhance pain management skills for them in Ethiopia.”

**Authors’ response:** **Thank you. We have removed the specified statement from the manuscript to ensure the content remains focused on the study’s actual objectives and scope.**

7. Remove this statement, “which initiates for designing appropriate strategies and intervention. Additionally, it reduces healthcare costs, hospitalization and improving quality of life for patients’ in Ethiopia.”

**Authors’ response:** **Thank you for your valuable comment. We have removed the statement**

Method

1. Please indicate in the text where the PRISMA checklist can be located.  (Supplementary file 1).

**Authors’ response:** **Thank you for your comment. We have added a reference to Supplementary File 1 where the PRISMA checklist can be found.**

2. "The review included all observational (cross-sectional) studies that were written in English.” Why only include cross-sectional studies? The second objective of this study is to identify associated factors. Hence, why did the authors exclude case control and other study designs? This previous question has not been answered. If there are case control studies on the topic that reported odds ratios, they should be included, as identifying factors is the second objective of this systematic review and meta-analysis.

**Authors’ response:** **We focused on cross-sectional studies because they are commonly used for assessing prevalence and are well-suited for examining the current state of pain management practices. However, we acknowledge that including case-control studies could have provided additional insights into factors associated with pain management practices. We have therefore expanded our inclusion criteria to also include case-control studies that reported odds ratios, in response to your feedback. Unfortunately we did not get primary studies which meet our criteria.**

3. Since this study included observational studies, please include the CoCoPop framework (condition, context, and population). It hasn’t been included in the revised manuscript. Please mention it in the manuscript in a clearer way.

**Authors’ response:** **Thank you for your feedback regarding the CoCoPop framework. We have now incorporated the CoCoPop framework into the revised manuscript as recommended. We have added a section to the Methods part of the manuscript that explains the CoCoPop framework and how it applies to our study. By saying we conducted a systematic review and meta-analysis of observational studies. To clarify the scope and focus of our review, we applied the CoCoPop framework, which outlines the Condition, Context, and Population relevant to our study.”(See at methods sections eligibility criteria, page number 6-7).**

4. “The third reviewer (WN) was involved while the differences between the data extractors persisted.” The word while should be replaced with when.

**Authors’ response: Thank you. We modified it. (See page number -7)**

5. On the data extraction, some pertinent variables are not extracted, such as population. Any reasons for not extracting the population? If there is no strong reason, I recommend including the population, as it will help in the subgroup analysis. The response provided for this question is not satisfactory. I personally believe that there might be differences in practice between different health professionals, for example, medical doctors vs. nurses. That is why I recommended extracting the population so that it can be considered for sub-group analysis.

**Authors’ response:** **we sincerely appreciate your thoughtful feedback and constructive suggestions.** **Reasons for not extracting population variables is due to that the most study subjects at primary studies were health care providers( nurses, midwifes, health officers, anesthesias, General practionier and specialists) which is difficult to study by sub-group analysis. But We acknowledge your point about potential differences in practice among different health professionals and we agree that population data could contribute to subgroup analysis, we tried to see by classifying my primary studies as Nurses only, nurses, midwifes and health officers as one and others (see page number15- 16 table 3).**

6. What type of method was used to conduct the sensitivity analysis? Please be specific and mention it. It hasn’t been mentioned. Please mention “one-at-a-time method” in the manuscript.

**Authors’ response:** **Thank you.** **The sensitivity analysis was performed using the "one-at-a-time method in the manuscript. We ensured to include this specific detail in the appropriate section to clarify the methodology used for sensitivity analysis.( see page number 9, line 1).**

7. The variables used for the sub-group analysis should be specifically mentioned.

**Authors’ response: Thank you.** **The variables included in the subgroup analysis are Method of pain management,** **Sample size,** **Types of pain, Years of publication and profession types. These variables were selected based on their clinical relevance and potential impact on the study outcomes (See page number -9, line 2)**

8. On the quality assessment, include all studies and cite them. Currently, only 30 out of the 38 studies are cited [13, 22-26, 38-61].

**Authors’ response:** **Thank you for your careful review of our manuscript.** **all studies are appropriately evaluated and cited**

9. Don’t include results regarding the quality assessment in the method section.

**Authors’ response:** **We revised the manuscript accordingly**

Results

1. Characteristics of the Included Studies: “The prevalence of health care providers’ pain management practice was obtained from thirty-eight included primary studies [13, 22-26, 38-61].” The authors mentioned 38 studies but cited only 30. Why? Please cite all studies.

**Authors’ response:** **Thank you. All 38 studies mentioned in the characteristics section are accurately cited.**

2. Most of the tables and figure captions are not self-explanatory. For example, Table 1’s caption is not self-explanatory. Please consider this example: Table 1: Characteristics of the included studies for the systematic review and meta-analysis of…The inception time for the included studies was 2015. But one study conducted in 2014 was included. Any justifications? The answer provided is not acceptable at all. Either include 2014 as an inception time or remove the study conducted in 2014.

**Authors’ response:** **Thank you once again for your insightful comments. We tried to improve the clarity of table and figure captions. And regarding to the inclusion of a study conducted in 2014, we updated the inception time to include studies from 2014.**

3. The description mentioned about the items of quality appraisal should be placed in the method section, not in the result section.

**Authors’ response:** **Thank you for your feedback.** **We ensured that this correction is made in our revised submission (See page number 8-10)**

4. Sensitivity Exploration: We conducted a sensitivity analysis to determine the impact of a specific study on the overall meta-analysis. The estimate from a single study is then closer to the pooled estimate, as seen by the forest plot, suggesting that there is no effect of a single study on the overall pooled estimate.” Statements explaining the methodology should not be included in the result section. Just show and clarify the results.

**Authors’ response:** **Thank you for your valuable feedback on our sensitivity analysis presentation. In our revised manuscript, we ensured to present the sensitivity analysis findings succinctly.**

5. In the sub-group analysis, there is no need to describe the importance and reasons for conducting a sub-group analysis.

**Authors’ response: Thank you we modified it according to you suggestions**

6. Words like “all most” (almost) are grammatically incorrect, and please use terms like 'similar’.

**Authors’ response:** **we corrected this mistake and replace it with the appropriate term**

7. For associated factors, most of the statements have grammatical errors, and additionally, the use of brackets varies from factor to factor. A P value of <0.000 is incorrect in the mathematical sense. Please replace it with <0.001.

**Authors’ response:** **Thank you for your thorough review and constructive feedback on our manuscript.** **We revised and replace instances of P < 0.000 with P < 0.001 throughout the document.**

-Discussion

1. The discussion section is still too shallow and it needs rewriting. You should discuss the variations in prevalence between this study and other studies in a detailed way and add other literature too.

**Authors’ response:** **Thank you for your feedback on the Discussion section.** W**e have made significant revisions to address these points But as far as our searching is concerned, there is limited of literatures.**

2. Factors should be discussed one by one.

**Authors’ response:** **Thank you for your feedback	. factors to pain management practice do not exist independently but are reciprocally linked and writing each one by one is a matter of repetition and also other reviewers were commented us to write collectively rather than individually. But we tried to rewrite and include the existing literatures.**

Limitation of the study

The limitations are not well stated and explained. Please explain the limitations in detail. You can consider the limitations mentioned by the included studies.

**Authors’ response:** **Your feedback is invaluable in helping us enhance, quality and clarity of our manuscript.** **In our revised version, we carefully expanded on the limitations, incorporating specific considerations mentioned by the included studies (See page number 22-23 at limitation section)**

Conclusion:

Please mention the comparators and the standard when saying low.

**Authors’ response:** **In our revised manuscript, we specified the comparators of a national target and standards used at conclusion section (See page number 23,line 11)**

Generally, even though there is a significant improvement in the revised manuscript, it still needs a significant revision. Additionally, despite the improvements, grammatical and typographical errors still persist. As a result, the manuscript needs language editing from an editorial service or from fluent English-speaking colleagues. Thank you!

**Authors’ response:** T**hank you for your thorough review and valuable feedback on our revised manuscript. We appreciate your acknowledgment of the improvements made and your critical assessment of the remaining issues, including grammatical, typographical errors. We understood the importance of ensuring clarity and precision in language, and we would take your recommendation seriously. Moving forward, we would seek professional language editing services to address the remaining grammatical and typographical errors effectively. Once again your feedback has been instrumental in guiding us toward further enhancing the quality of our manuscript.**
